# Sulfonamide Inhibition Studies of an α-Carbonic Anhydrase from *Schistosoma mansoni*, a Platyhelminth Parasite Responsible for Schistosomiasis

**DOI:** 10.3390/ijms21051842

**Published:** 2020-03-07

**Authors:** Andrea Angeli, Mariana Pinteala, Stelian S. Maier, Bogdan C. Simionescu, Akram A. Da’dara, Patrick J. Skelly, Claudiu T. Supuran

**Affiliations:** 1 Dipartimento Neurofarba, Sezione di Scienze Farmaceutiche e Nutraceutiche, Università degli Studi di Firenze, Via U. Schiff 6, 50019 Sesto Fiorentino, Florence, Italy; andrea.angeli@unifi.it; 2 Centre of Advanced Research in Bionanoconjugates and Biopolymers Department, “Petru Poni” Institute of Macromolecular Chemistry, 700487 Iasi, Romania; pinteala@icmpp.ro (M.P.); smaier@ch.tuiasi.ro (S.S.M.); bcsimion@icmpp.ro (B.C.S.); 3 Polymers Research Center, Polymeric Release Systems Research Group, “Gheorghe Asachi” Technical University of Iasi, 700050 Iasi, Romania; 4 Department of Infectious Disease and Global Health, Cummings School of Veterinary Medicine, Tufts University, North Grafton, MA 01536, USA; Akram.Da_darah@tufts.edu (A.A.D.); Patrick.Skelly@tufts.edu (P.J.S.)

**Keywords:** carbonic anhydrase, sulfonamide, schistosomiasis, *Schistosoma mansoni*, inhibitor, trematode, blood fluke

## Abstract

Schistosomiasis is a debilitating infection provoked by parasitic flatworms called schistosomes. The species *Schistosoma mansoni* is endemic in Africa, where it causes intestinal schistosomiasis. Recently, an α-carbonic anhydrase (CA, EC 4.2.1.1) was cloned and characterized from this organism and designated as SmCA. The protein is expressed in the tegument (skin) of *S*. *mansoni* at the host–parasite interface. Recombinant SmCA possesses high catalytic activity in the CO_2_ hydration reaction, similar to that of human CA isoform II with a k_cat_ of 1.2 × 10^6^ s^−1^ and a k_cat_/K_M_ of 1.3 × 10^8^ M^−1^·s^−1^. It has been found that schistosomes whose SmCA gene is suppressed using RNA interference are unable to establish a robust infection in mice, suggesting that the chemicals that inhibit SmCA function should have the same debilitating effect on the parasites. In this study, a collection of aromatic/heterocyclic sulfonamides were investigated as possible SmCA inhibitors. Several sulfonamides inhibited SmCA with medium to weak potency (K_I_ values of 737.2 nM−9.25 μM), whereas some heterocyclic compounds inhibited the enzyme with K_I_ values in the range of 124−325 nM. The α-CA from *S. mansoni*, SmCA, is proposed as a new anti-schistosomiasis drug target.

## 1. Introduction

Carbonic anhydrases (CAs, EC 4.2.1.1) are ubiquitous metallo-enzymes present in all kingdoms of life [1,2,3]. To date, eight distinct and genetically independent CA classes are known, and these are classified as α-, β-, γ-, δ-, ζ-, η-, θ and, ι- isoforms [4,5,6,7,8]. This superfamily of enzymes catalyzes the same physiologically reversible reaction: the conversion of carbon dioxide (CO_2_) to bicarbonate (^−^HCO_3_) and protons (H^+^) [9]. This important physiological reaction plays a key role in several biosynthetic pathways, such as the biosynthesis of fatty acids, amino acids and nucleotides [10,11,12]. In recent years, CAs have been investigated in detail in pathogens such as bacteria and protozoa [13,14,15,16]. One aim of such work is the search for new antibiotics and anti-infectives that exhibit novel mechanisms of action [17,18,19,20]. In this context, schistosomiasis, a tropical disease caused by trematode parasites of the genus *Schistosoma*, has been neglected. The disease is considered to be the second most debilitating parasitic infection in terms of morbidity and mortality, after malaria [21,22]. Indeed, schistosomes are estimated to currently infect more than 206 million people worldwide, and over 700 million people live in areas where the disease is endemic [23]. Schistosomiasis is prevalent in tropical and subtropical areas, particularly in poor communities that lack access to safe drinking water and adequate sanitation. The current treatment for infection is centered on the drug praziquantel [24]. However, the exclusive use of praziquantel has been accompanied by reports in the literature that parasites isolated from some patients have been resistant to treatment [25,26]. The possibility of developing new anti-schistosomiasis agents with a novel mechanism of action has come to the fore in light of the fact that an α-CA isoform designated as SmCA has recently been identified and characterized from *S. mansoni*. SmCA is a ~37 kDa GPI-linked glycoprotein that is expressed in the tegument (skin) of *S. mansoni* at the host–parasite interface [19]. Such proteins are considered as good drug targets given their accessibility—they are foundon the surface of worms. The SmCA gene is expressed in all schistosome life stages examined, with the highest relative expression seen in adult male worms [19]. Schistosomes whose SmCA gene is suppressed using RNAi have been found to be unable to establish a robust infection in mice [19]. This suggests that chemicals that inhibit SmCA function will have the same debilitating effect on the parasites and could curtail infection [19]. In this work, a collection of 24 aromatic/heterocyclic sulfonamide compounds were tested for their ability to block the enzymatic action of recombinant SmCA (that has been produced and purified from CHO-S cells) [19]. The long-term aim of this work is to identify potent SmCA-blocking compounds that can incapacitate schistosomes and cure infection.

## 2. Results

The SmCA catalytic activity for the CO_2_ hydration reaction is shown in Table 1; data for other CAs, such as the widespread and well-investigated human (h) isoforms hCA I and hCA II, as well as the *Trypanosoma cruzi* enzyme (TcCA, belonging to the α-class [27]), and a β-CA from *Leishmania donovani* (LdCA) [28], are included for comparison. A stopped-flow CO_2_ hydrase assay was used to measure the catalytic activity of these enzymes under identical conditions [29].

SmCA has kinetic parameters similar to human isoform hCA II, one of the most catalytically active enzymes known [3]. Indeed, with a kcat/Km of 1.3 × 10^8^ M^−1^·s^−1^, SmCA has basically indistinguishable kinetic parameters compared to both hCA II and to the protozoa α-CA TcCA (Table 1). All three are much more active as a catalyst for CO_2_ hydration compared to hCA I or the β-class CA, LdCA. However, SmCA is much less sensitive to the sulfonamide inhibitor acetazolamide (5-acetamido-1,3,4-thiadiazole-2-sulfonamide,**AAZ**) compared to all the other CAs tested. 

**Figure 1:**
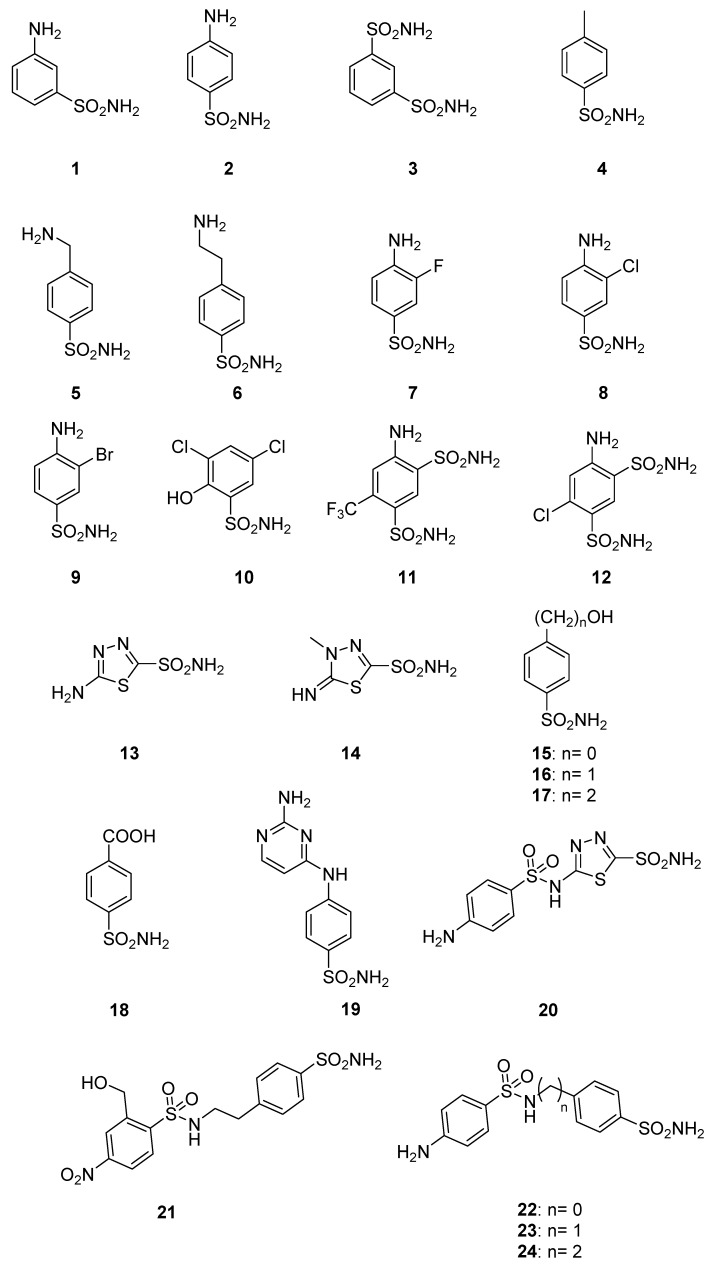
Structures **1**−**24**.

Sulfonamides are the main class of zinc-binding CA inhibitors (CAIs), [2,3] but several other classes of inhibitors have also been reported recently, such as the thiols, dithiocarbamates, coumarins, polyamines and selenols among others [30,31]. We have thus included a range of sulfonamides in an initial screening program to find the compounds targeting this new protozoan enzyme. Sulfonamides, thiols, dithiocarbamates and selenols possess a similar mechanism of action, as they bind to the zinc ion within the active site cavity and substitute the non-protein zinc ligand (the hydroxide ion/water molecule) [2,3,30,31]. Here, we investigated a panel of 24 sulfonamides (compounds **1−24**, whose molecular structure is depicted in Figure 1) for their ability to inhibit recombinant SmCA (rSmCA). Table 2 presents the results of this analysis: the data for SmCA (highlighted in green) are compared to those generated with human isoforms hCA I and hCA II and the CAs from the protozoan parasites *T. cruzi* (TcCA) and *L. donovani* (LdCA).

The sulfonamides **1−24** and **AAZ** investigated in this study were either commercially available or prepared in the manner that was reported earlier by our group [27,28].

Several of the simple sulfonamides investigated here, such as compounds **1**, **2**, **4–7** and **10**, were weakly inhibitory, with K_I_ values of 3.1–9.5 µM. Note that all these compounds are aromatic derivatives with incorporation of an amine group (Figure 1). More effective inhibition of SmCA is observed with the remaining compounds with primary amine groups such as **8**, **9**, **11** and **12** with K_I_ values in the range 137−807 nM (Table 2). Note that these compounds belong to a rather heterogeneous class of aromatic sulfonamides, so the structure−activity relationship (SAR) is not straightforward. On the other hand, the heterocycle compounds **13**, **14** and **20** were better inhibitors than the benzenesulfonamide scaffold, with K_I_ spanning between 124.2−325.1 nM.

Modifications to the tail moiety of the inhibitors **15–17** and **22–24** followed the "tail approach", which involves modifying the tails of the well-known aromatic sulfonamide scaffolds in order to modulate the physicochemical properties, such as the water solubility and enzyme-binding capacity of these pharmacological agents. Indeed, the tail length of the compounds **15–17** is optimal for derivative **17** with an ethyl chain (K_I_ 605.6). The inhibition activity of the compounds **22–24** exhibited the same inhibition feature, showing the best activity with an ethyl chain (**24**) with K_I_ of 183.5 nM. While the inhibition profile of the schistosome enzyme SmCA was very different from that of the mammalian enzymes hCA I and hCA II, none of the tested compounds showed greater inhibition of the worm enzyme when compared to both human isoforms (Table 2).

## 3. Materials and Methods 

### 3.1. General

Compounds **1–24** were commercially available, high-purity reagents from Sigma-Aldrich, Milan, Italy. SmCA, TcCA and LdCA were recombinant proteins produced using the methods previously reported by our groups [19,27,28].

### 3.2. Carbonic Anhydrase Assay

A stopped-flow method [29] was used to assay CA-catalyzed CO_2_ hydration activity with Phenol red as an indicator, working at the absorbance maximum of 557 nm, following the initial rates of the reaction for 10–100 s. For each inhibitor, at least six traces of the initial 5–10% of the reaction were used to determine the initial velocity (see Supplementary Materials). The uncatalyzed rates were determined in the same manner and subtracted from the total observed rates. Stock solutions of inhibitor (0.01 mM) were prepared in distilled-deionized water with 5% DMSO and dilutions up to 0.1 nM were performed thereafter with a buffer. The inhibition constant (K_I_) of each tested compound was obtained by considering the classical Michaelis–Menten equation, which was fitted by non-linear least squares using PRISM 3 software.

### 3.3. Expression and Purification of Recombinant SmCA

The full-length coding sequence of SmCA (GenBank accession number, MK611932), including the predicted signal peptide and glycophosphatidylinositol (GPI) anchor domain, was codon optimized using hamster codon preferences and synthesized commercially (Genscript). Next, the region encoding the amino acids N21–A298 (i.e., lacking the amino terminal signal peptide and the carboxyl terminal GPI-anchoring signal) was generated by polymerase chain reaction (PCR) using forward and reverse primers containing AscI and XhoI restriction sites, respectively, and the synthetic codon-optimized gene as a template. The amplified product was cloned into the pSecTag2A plasmid (Invitrogen, Waltham, MA, USA) at the AscI and XhoI sites in frame with the Igκ leader sequence at the 5’-end and a myc epitope and 6-histidine tag at the 3’-end. Successful in-frame cloning was confirmed by sequencing at the Tufts University Core Facility. To express recombinant SmCA (rSmCA), suspension-adapted Free-Style Chinese Hamster Ovary Cells (CHO-S) were transfected with plasmid using the Free-Style Max Reagent following the manufacturer’s instructions (Invitrogen). Cells were harvested at various time points post-transfection to monitor viability (by the trypan blue exclusion test) and rSmCA expression (by Western blotting). To facilitate protein production, stable cell line clones secreting rSmCA were selected by treating transfected cells with 250 μg/mL of Zeocin for two weeks; individual clones that produced high yields (5–10 mg) of purified active rSmCA/L were maintained. Recombinant SmCA was purified from the cell culture medium by standard Immobilized Metal Affinity Chromatography (IMAC) using HisTrap™ Excel columns, following the manufacturer’s instructions (GE Healthcare Life Sciences, Chicago, IL, USA). Purified recombinant protein, eluted from the column, was dialyzed overnight at 4 °C against 50mM Tris–HCl (pH 7.4), 150mM NaCl, then concentrated by ultrafiltration centrifugation (Pierce Protein Concentrators, 10 K MWCO, Thermo Scientific, Waltham, MA, USA). Final protein concentration was determined using a BCA Protein Assay Kit (Pierce, Waltham, Massachusetts, USA). Aliquots of eluted protein were resolved by 4–20% SDS-PAGE to assess purity, and some were tested for specificity by Western blotting using anti-myc tag and anti-SmCA antibodies.

## 4. Conclusions

We report the inhibition profile of the α-CA from the platyhelminth parasite *S. mansoni*, a causative agent of schistosomiasis. This enzyme, SmCA, showed a high catalytic activity for the CO_2_ hydration reaction, being similar kinetically to α-CA from the protozoan *T. cruzi* and the human isoform hCA II. A library of aromatic/heterocyclic sulfonamides were investigated as possible SmCA inhibitors. The aromatic sulfonamides were, generally, weak inhibitors (K_I_ values of 737.2 nM–9.25 μM), whereas some heterocyclic compounds inhibited this enzyme, with K_I_ values in the range of 124.2–325.1 nM. However, no compounds were identified in this chemical screen that preferentially inhibited SmCA to a greater degree than the human CAs. The overall inhibition profile of the schistosome enzyme differed substantially from those of the human isoforms tested, and this highlights the different biochemistries of the parasite versus the host enzymes. These results are not unexpected given that crystal structure comparisons of the worm versus the human enzymes has revealed differences in the active sites (and other regions) of the proteins [19]. These differences suggest that it will be possible to identify chemicals that selectively and potently block SmCA action while exerting little or no inhibition of human homologs. Indeed, a number of compounds (phenylarsonic acid, phenylbaronic acid, sulfamide) have been shown to exhibit substantially more favorable K_I_s for SmCA versus the human isoforms [19]. Thus, we believe that SmCA is an important target for developing anti-parasitic trematode drugs that exert a novel mechanism of action.

## Figures and Tables

**Table 1:** Kinetic parameters for the CO_2_ hydration reaction catalyzed by platyhelminth SmCA (green row) as well as the α-CA isozymes of human (h) hCA I and hCA II, protozoan *Trypanosoma cruzi* enzyme (TcCA) and *Leishmania donovani* (LdCA) at 20 °C, pH 7.5 (for the α-CAs) and pH 8.4 (for the β-CA). Inhibition data (K_I_ values) generated with the clinically used drug Acetazolamide (**AAZ**, 5-Acetamido-1,3,4-thiadiazole-2-sulfonamide) are shown in the right-hand column.

Enzyme	k_cat_ (s^−1^)	K_m_ (M)	k_cat_/K_m_ (M^−1^·s^−1^)	K_I_ (AAZ) (nM)
hCA I ^a^	2.00 × 10^5^	4.0 × 10^−3^	5.00 × 10^7^	250.0
hCA II ^a^	1.40 × 10^6^	9.3 × 10^−3^	1.50 × 10^8^	12.1
TcCA ^b^	1.21 × 10^6^	8.1 × 10^−3^	1.49 × 10^8^	61.6
LdCA ^c^	9.35 × 10^5^	15.8 × 10^−3^	5.9 × 10^7^	91.7
SmCA ^d^	1.2 × 10^6^	9.2 × 10^−3^	1.3 × 10^8^	42.5

All experiments employed recombinant isozymes and used the stopped-flow CO_2_ hydrase assay method ^a^ [3,4], ^b,c^ [27,28], ^d^ [19].

**Table 2:** Inhibition of *S. mansoni* SmCA (green column) compared with human carbonic anhydrase isoforms hCA I and hCA II, as well as protozoan CAs from *T. cruzi* (TcCA) and *L. donovani* (LdCA), using sulfonamides **1−24** and as assessed by the stopped-flow CO_2_ hydrase assay [29].

K_I_ (nM) *					
Inhibitor	hCA I ^a^	hCA II ^a^	SmCA ^b^	TcCA ^c^	LdCA ^d^
**1**	45400	295.0	8423 ± 177	25460	5960
**2**	25000	240.0	6819 ±1 50	57300	9251
**3**	28000	300.0	915.6 ± 23.8	63800	8910
**4**	78500	320.0	4534 ± 90	44200	>10000
**5**	25000	170.0	9558 ± 191	7231	>10000
**6**	21000	160.0	7242 ± 144	9238	>10000
**7**	8300	60.0	3190 ± 70	8130	15600
**8**	9800	110.0	737.2 ± 19.1	6925	9058
**9**	6500	40.0	807.7 ± 16.9	8520	8420
**10**	6000	70.0	9268 ± 185	9433	9135
**11**	5800	63.0	183.1 ± 9.1	842	9083
**12**	8400	75.0	644.8 ± 19.9	820	4819
**13**	8600	60.0	137.4 ± 6.5	534	584
**14**	9300	19.0	325.1 ± 6.5	652	433
**15**	6.0	2.0	758.2 ± 26.5	73880	927
**16**	164.0	46.0	1462 ± 32.1	71850	389
**17**	185.0	50.0	605.6 ± 21.3	66750	227
**18**	109.0	33.0	820.7	84000	59.6
**19**	95.0	30.0	1831 ± 36.6	810	>10000
**20**	690.0	12.0	124.2 ± 5.9	88.5	95.1
**21**	55.0	80.0	163.2 ± 6.5	134	50.2
**22**	21000	125.0	550.4 ± 14.8	365	136
**23**	23000	133.0	523.7 ± 15.7	243	87.1
**24**	24000	125.0	183.5 ± 4.5	192	73.4
**AAZ**	250.0	12.1	42.5	61.6	91.7

* The data represents the mean of three different assays; the mean errors are ± 2–5% of the reported values. All experiments employed recombinant isozymes and used the stopped-flow CO_2_ hydrase assay method ^a^ [3,4]. ^b,c^ [26,27], ^d^ [19].

## Supplementary Materials

The following are available online at https://www.mdpi.com/1422-0067/21/5/1842/s1, Carbonic Anhydrase activity.

## Abbreviations

CA	Carbonic Anhydrase
CAI	Carbonic Anhydrase Inhibitors
SmCA	Schistosoma Mansoni Carbonic Anhydrase
DMSO	Dimethylsulfoxide
