# Supplementary material for: Sulfonamide Inhibition Studies of an α-Carbonic Anhydrase from Schistosoma mansoni, a Platyhelminth Parasite Responsible for Schistosomiasis
Source: Int J Mol Sci. 2020 Mar 7;21(5):1842. doi: 10.3390/ijms21051842 (PMC7084386; doi:10.3390/ijms21051842)

Supplementary Information for

Sulfonamide Inhibition Studies of an α-Carbonic Anhydrase from *Schistosoma mansoni*, a Platyhelminth Parasite Responsible for Schistosomiasis

*Andrea Angeli[1,2], Mariana Pinteala[2], Stelian S. Maier[2,3], Bogdan C. Simionescu[2], Akram. A. Da'dara[4], Patrick J. Skelly[4], Claudiu T. Supuran[1]**

[1] Università degli Studi di Firenze, Dipartimento Neurofarba, Sezione di Scienze Farmaceutiche e Nutraceutiche, Via U. Schiff 6, 50019 Sesto Fiorentino, Florence, Italy; andrea.angeli@unifi.it (A.A.)

[2] Centre of Advanced Research in Bionanoconjugates and Biopolymers Department,"Petru Poni" Institute of Macromolecular Chemistry, Iasi, Romania; pinteala@icmpp.ro (M.P.), smaier@ch.tuiasi.ro (S.S.M.), bcsimion@icmpp.ro (B.C.S.)

[3] Polymers Research Center, Polymeric Release Systems Research Group, "Gheorghe Asachi" Technical University of Iasi, Iasi, Romania; smaier@ch.tuiasi.ro (S.S.M.)

[4] Department of Infectious Disease and Global Health, Cummings School of Veterinary Medicine, Tufts University, North Grafton, MA, USA; Akram.Da_darah@tufts.edu (A.A.D.), Patrick.Skelly@tufts.edu (P.J.S.)

[*] Correspondence: claudiu.supuran@unifi.it; (C.T.S.)

Contents

1. *S. mansoni* Carbonic Anhydrase activity *S3*

1. *S. mansoni* Carbonic Anhydrase activity:

Compound 1:

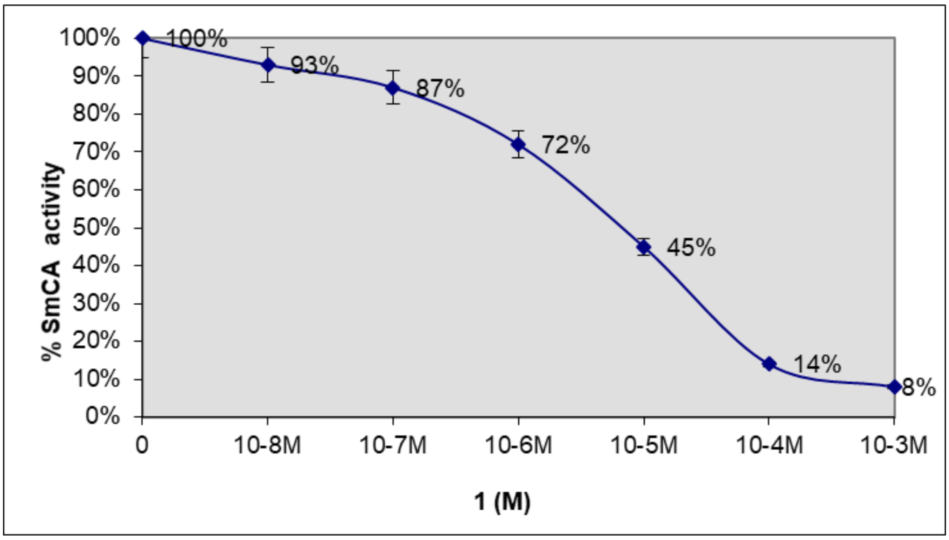

Compound 2:

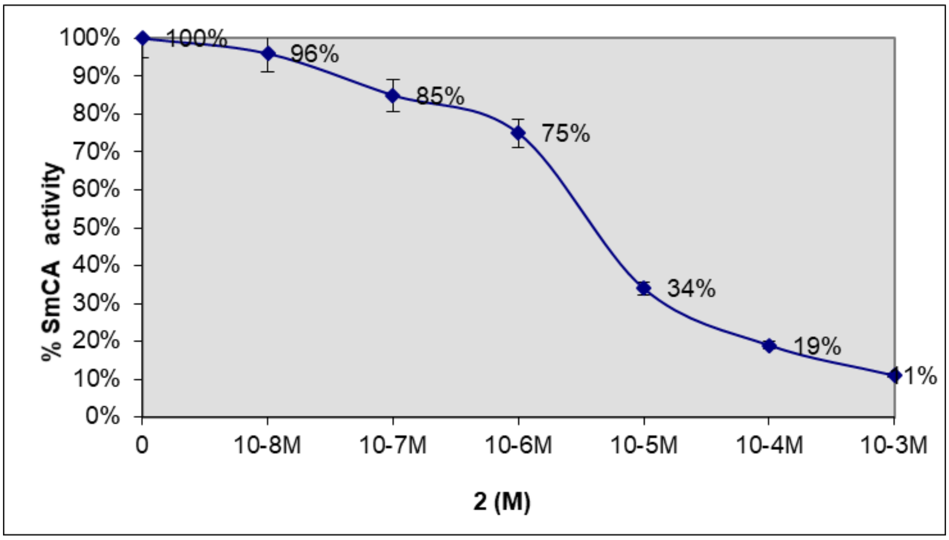

Compound 3:

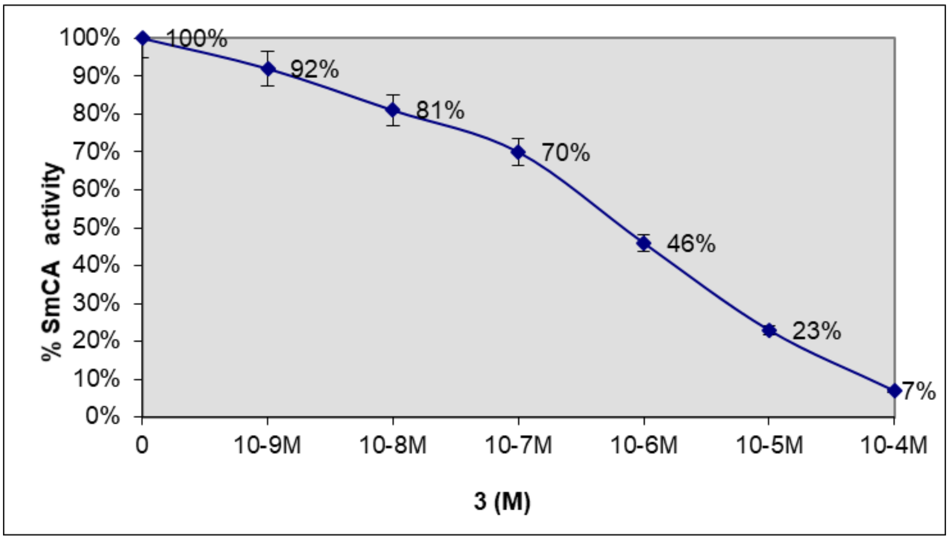

Compound 4:

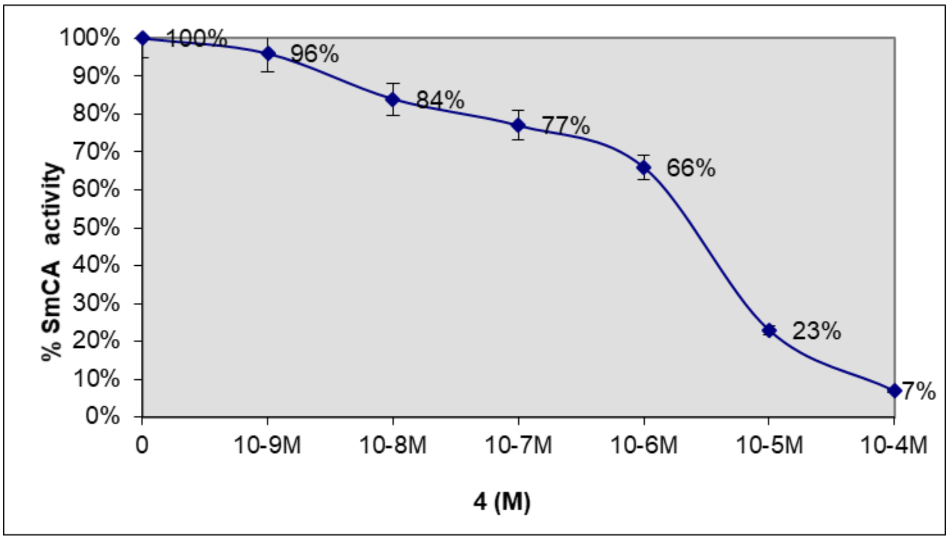

Compound 5:

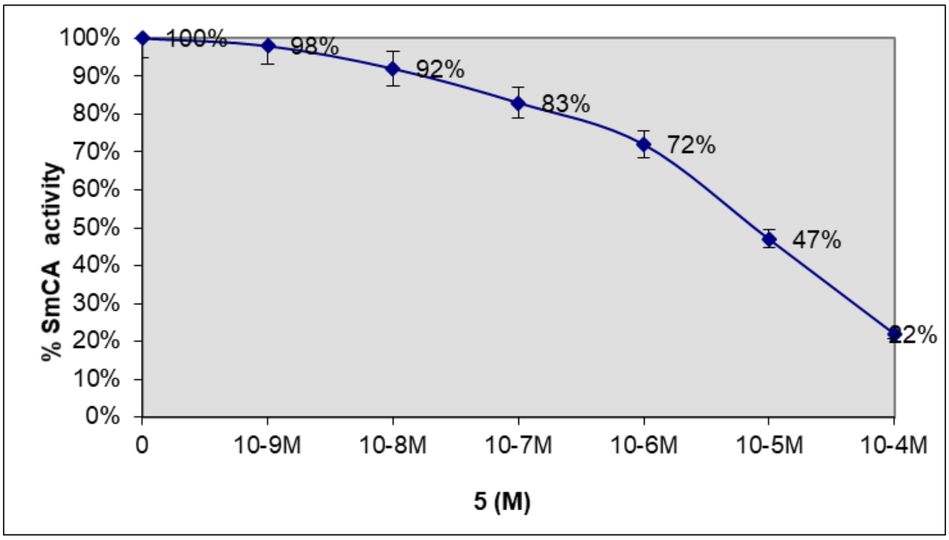

Compound 6:

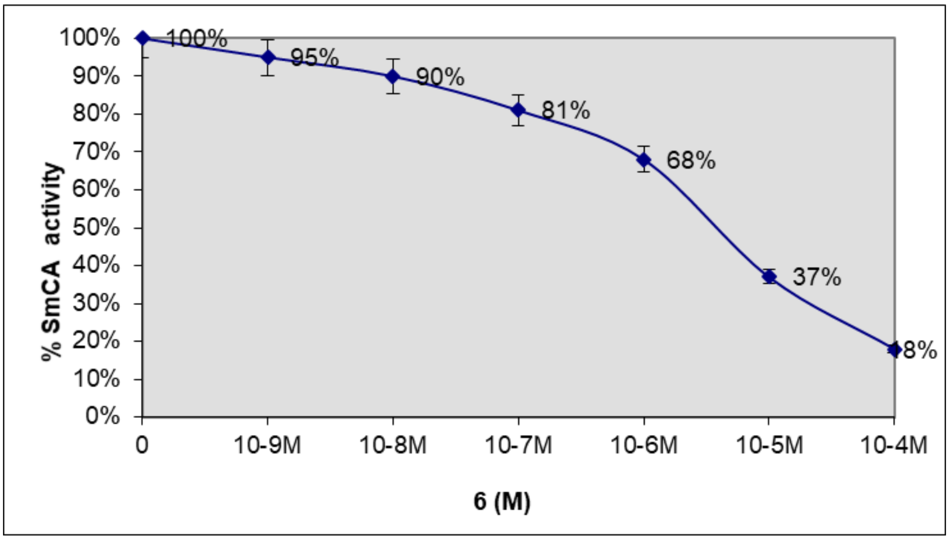

Compound 7:

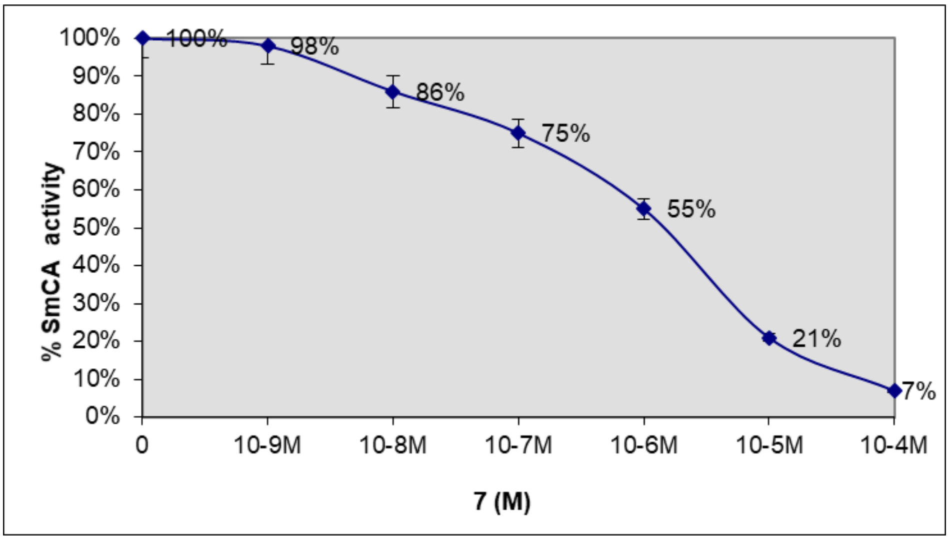

Compound 8:

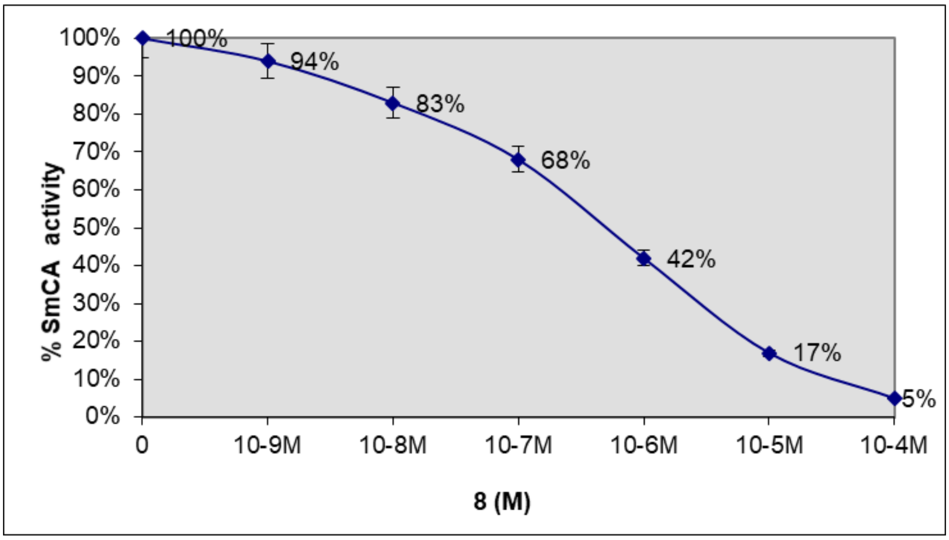

Supplement: Supplementary file 1 [file ijms-21-01842-s001.pdf]
